# Quantitative Lateral Flow Assays for Salivary Biomarker Assessment: A Review

**DOI:** 10.3389/fpubh.2017.00133

**Published:** 2017-06-14

**Authors:** Olga Miočević, Craig R. Cole, Mary J. Laughlin, Robert L. Buck, Paul D. Slowey, Elizabeth A. Shirtcliff

**Affiliations:** ^1^Iowa State University, Ames, IA, United States; ^2^Oasis Diagnostics^®^ Corporation, Vancouver, WA, United States

**Keywords:** salivary cortisol, salivary biomarkers, rapid biomarker assessment, point-of-care, rapid diagnostic tests, lateral flow immunoassays

## Abstract

Saliva is an emerging biofluid with a significant number of applications in use across research and clinical settings. The present paper explores the reasons why saliva has grown in popularity in recent years, balancing both the potential strengths and weaknesses of this biofluid. Focusing on reasons why saliva is different from other common biological fluids such as blood, urine, or tears, we review how saliva is easily obtained, with minimal risk to the donor, and reduced costs for collection, transportation, and analysis. We then move on to a brief review of the history and progress in rapid salivary testing, again reviewing the strengths and weaknesses of rapid immunoassays (e.g., lateral flow immunoassay) compared to more traditional immunoassays. We consider the potential for saliva as an alternative biofluid in a setting where rapid results are important. We focus the review on salivary tests for small molecule biomarkers using cortisol as an example. Such salivary tests can be applied readily in a variety of settings and for specific measurement purposes, providing researchers and clinicians with opportunities to assess biomarkers in real time with lower transportation, collection, and analysis costs, faster turnaround time, and minimal training requirements. We conclude with a note of cautious optimism that the field will soon gain the ability to collect and analyze salivary specimens at any location and return viable results within minutes.

## Introduction

This paper examines emerging salivary technologies as worthwhile alternatives to traditional blood and serum based systems and highlights novel innovations in the field for rapid, point-of-care (POC) salivary testing.

Within the clinical setting, biomarkers are typically analyzed from blood, serum, urine, tears, tissue, breath, or stool specimens. In research settings, specimen collection and analysis is more limited, yet still often involves blood collection. Collection and analysis of blood samples can be invasive and stressful to the donor, creates challenges for repeat collection, and may require extensive collection equipment to comply with biosafety precautions, while also being costly to process. Saliva has emerged within the past two decades as a viable biofluid because it reduces many of the drawbacks of invasive collection with minimal reduction in sample efficacy. In the case of hormone biomarker assessment, there are further advantages related to the use of saliva since it is known to reflect the free or biologically active hormone fraction, whereas blood may detect levels of the bound or complexed hormone.

The present paper reviews the strengths and weaknesses of salivary biomarker assessment with an emphasis on hormonal biomarkers and considers the potential impact of new directions in biomarker assessment on research and practice. The paper also highlights the utility of near real-time assessment and describes how lateral flow immunoassay (LFIA) can be used to obtain results quickly and efficiently. The goal of this paper is to then combine these two technological advancements, LFIA rapid assessment, and salivary testing, with explicit consideration of the advantages and disadvantages as well as the challenges and opportunities for near real-time salivary diagnostics. We focus on rapid salivary assessment of biomarkers such as cortisol and other small molecule analytes as part of an emerging methodology with a high future contribution to the immunoassay field.

## Saliva as an Accessible Biological Fluid

### Rethinking the Tradition of Blood and Other Fluids

Serum or plasma biomarkers are traditional “gold standards” for hormone collection, although they each suffer from certain drawbacks. Collection and analysis of biofluids such as blood can be costly, time consuming, and invasive to individuals providing samples as well as professionals analyzing the specimens. Venipuncture can induce a stress response, as shown by multiple laboratory studies (1, 2), which can raise concerns for studies focused on determination of stress-related biomarkers (e.g., cortisol) or who work with populations in which a stress response may be ill-advised (e.g., Addison’s disease) or challenging (e.g., children and neonates (3)). The invasiveness of blood draw protocols also limits the ability of researchers to carry out multiple biofluid collections, for instance in studies that require multiple samples or longitudinal samples. Moreover, there are inherent challenges in specific settings where venipuncture is difficult or where biosafety precautions and blood-borne pathogen exposure are difficult to minimize. The potential for pathogen exposure is, unfortunately, always present. Handling blood requires a highly trained phlebotomist. Lastly, a significant drawback of blood-based biomarker analysis is the cost and time required for safe transportation of specimens. Disposal of these specimens requires extensive biohazardous waste procedures, adding further expense. The present paper does not argue that blood lacks utility, but rather suggests that there are protocols in which the “gold standard” biofluid may not ultimately be the preferred collection method.

Notably, biofluids other than blood are often used for assessment of biomarkers in research and clinical settings. Urine specimens are most often associated with home pregnancy or fertility tests, but are also used for detection of renal disease and injury (4, 5), prostate cancer (6, 7), assessment of hormones such as growth hormone abuse in sports (8) and detection of the “cuddle hormone,” oxytocin (9, 10), as well as drug testing (11, 12). Urine testing has the advantage of being non-invasive, is available in large quantities, and its use spans across detection of disease and large molecule analytes (e.g., oxytocin). Similar to other biofluids, urine specimens require specific training for certain collection methods (i.e., clean catch or catheter), storage, and analysis practices. Importantly, urine specimens should be collected *via* clean catch method, in order to avoid contamination. Furthermore, specimens can be difficult to obtain if patients are unable to produce urine and often samples can be tampered with (e.g., in drug testing).

Breath specimens are collected as exhaled breath condensate or exhaled breath vapor (EBV), and can be used for detection of volatile organic compounds [VOCs (13)]. VOC analysis has facilitated extensive research on the detection of compounds related to many medical conditions such as lung infections and obstructive pulmonary disease, breast and lung cancers, as well as asthma and diabetes (13). Breath collection can be challenging, because of the importance of capturing clean specimens for better detection of endogenous VOCs (13). There are many techniques for collection and analysis of breath specimens, however, reviewing them is beyond the scope of this paper [see Ref. (13) for a detailed discussion].

Tears are also used for the non-invasive detection of diseases. For example, human tear specimens are used for analysis of proteins in diabetics (14), as well as measurement of biomarkers of ocular and systemic diseases (15). Tear specimens can be collected using the Schirmer’s test (16), glass capillary micropipettes, or porous polyester rods (17), and other collection methods (18).

It is beyond the scope of this paper to exhaustively review the available methods and applications for all testable biofluids. Commensurate with this Research Topic’s scope, we focus our attention on saliva as a viable alternative to the presumed gold standard, blood. Below we present the strengths and weaknesses of this biofluid.

### Emergence of Saliva As a Viable Alternative

The opportunity to evaluate biomarkers in saliva as a result of new isolation and downstream testing technologies has provided new opportunities in research and study protocols that are not possible using invasive collection methods. Saliva has been increasingly appreciated as a readily available biofluid in laboratory testing for bacteria (19, 20), systemic diseases (21), genomics (22), drugs of abuse (23), hormones (24), and a growing number of other health indicators [e.g., Ref. (25)].

Consider, for example, how research with salivary cortisol has blossomed extensively since it was first measured in the early 1970s. The number of published articles has grown exponentially for the past three decades with 964 salivary cortisol articles published in peer-reviewed journals in a single year alone (2016 according to Harzing’s publish-or-perish, 4/22/2017). In one fiscal year, the National Institutes of Health (NIH) funded over $19 million in research with salivary cortisol (according to NIH RePorTer query for cortisol and saliva, 4/22/2017). Below, we consider some of the potential reasons for this burgeoning interest in saliva, with special attention given to salivary cortisol.

### Advantages of Saliva

In addition to serving multiple functions including chewing and swallowing, saliva contains information about the physiology of the body. It is often referred to as a “mirror of the body” and similar to blood, saliva contains proteins, and DNA and RNA moieties, which can be evaluated for the identification of abnormalities and diseases. Saliva offers many benefits as an investigative biofluid beyond the three briefly described here [see Ref. (26)].

First, methods of saliva collection are *non-invasive*. Saliva is easily accessible, allowing for repeated measurements without the stress associated with drawing blood *via* venipuncture. It is feasible and common to collect repeated samples of saliva over time and thus to track how the biomarkers of interest change or respond to an experimental protocol without requiring repeated venipuncture, finger sticks, or an intravenous catheter. Common saliva collection methods include pooling of unstimulated passive drool directly into a tube, use of cotton or absorbent pads and swabs, or suction devices for extraction of sample directly from the salivary glands. With minimal risk or harm to donors, saliva is readily available in adequately large volumes. Saliva is an ideal biofluid for collection and testing in vulnerable populations, diverse populations for whom saliva specimens may be preferential to blood, and even for populations where cultural religious practice prohibits blood sampling. In addition, when handling salivary specimens, there is minimal risk of contracting infections from blood-borne pathogens such as the human immunodeficiency virus (HIV) or hepatitis, which is a potential hazard when working with other biofluids, particularly blood (27). These particular characteristics have allowed salivary diagnostics to be increasingly used in the detection of viral and bacterial pathogens, as well as diagnosis of rubella, Ebola, and HIV (28).

Second, saliva is easily collected in POC contexts. The ability of biomarker measurement to be reflective of the health of an individual is most apparent when the biomarker reflects the social context of the individual at that time. The fact that saliva samples are fairly easy to collect, store, and ship has precipitated a significant interest and growth in the use of salivary testing, for example in developing countries, remote sites, or changing locations (e.g., roadside drug testing, consumer genetics, wellness testing). The POC advantage has also opened a window of opportunity for collecting specimens in naturalistic environments such as in the privacy of a participant’s home, school, geriatric clinic, mobile van, or field-based study location. For example, we have published studies that have involved saliva collection at a roller coaster theme park, a haunted house, a karaoke bar, a rugby match, and a soccer game (29). Given that many biomarkers show circadian fluctuations, the advantage of POC testing has allowed saliva collection to be performed at times of day in which clinical visits or venipuncture would be unlikely or would require extended hospital stays (e.g., first thing in the morning, bedtime, or throughout the evening).

Third, as saliva collection has become more widespread, it is increasingly recognized as a *biofluid with unique properties* in and of itself. This potential benefit lies in contrast to a traditional view of saliva as an alternative to blood and for which a high serum–saliva correlation is essential. The oral cavity is viewed as the entrance to the gastrointestinal tract and, as a result, saliva collection is beginning to be recognized as a “gateway” for physiological information. The oral cavity is colonized by living organisms and bacteria, and thus salivary biomarkers capture the first integration of the individual’s physiology through their microbiome. In addition to being a standard biofluid for DNA or RNA collection (e.g., cheek swabs, buccal scrapes, whole saliva by expectoration), cutting-edge research is also beginning to view mRNA and salivary gene expression indices, collectively known as the transcriptome, within saliva as informative for health (30, 31). Lastly, the oral cavity is a hot-spot for immune activation. Secretory IgA, an immunoglobulin specific to saliva, provides non-specific information about the immune function in the oral cavity. In addition to being informative about periodontal disease, secretory IgA serves as a marker of oral inflammation, which can exert an overall system inflammatory response with long-term impact on health (32). sIgA has also been used for diagnosis of dengue viral infection (33) and is a heavily used biomarker in the field of sports medicine (34–36). Other oral immune metrics take advantage of the presence of viral infection (e.g., herpes simplex virus) and bacterial activation (e.g., streptococcus) in saliva (37). These extend to functional immune measures, which capture how well an individual’s saliva itself fights an oral pathogen to restore health (38). In addition, in the case of hormone biomarker assessment, a major advantage related to the use of saliva is its ability to reflect the free or biologically active hormone fraction (39), whereas blood may detect levels of the bound or complexed hormone.

### Disadvantages/Challenges of Saliva

Although saliva appears to be a great alternative to sample matrices such as blood and urine, it has disadvantages and challenges. Saliva is a heterogeneous biofluid, produced by numerous minor salivary glands in addition to the three main salivary glands: parotid, submandibular, and sublingual (40). It consists mostly of water and is produced in large quantities every day, by the average human being (41). The small fraction of saliva, which is not water contains a multitude of molecules, bacteria, and viruses. The salivary glands are surrounded by capillaries, allowing for exchange of molecules between blood and saliva. Specific constituents such as drugs and hormones, often reach saliva through passive diffusion and ultrafiltration (42), but some biomarkers enter through active transport. Saliva’s unique properties as a biofluid need to be properly considered in order to obtain optimum results.

Perhaps the most important drawback of saliva is that concentrations of specific biomarkers in saliva are often much lower (10–1,500 times lower) than in plasma (43) due mainly to the fact that saliva is an ultrafiltrate of blood and consists mostly of water. This limitation has been addressed through recent advances in instrumentation for detection of biomarkers that includes highly sensitive technologies such as ultra-sensitive ELISAs, mass spectrometry, next generation sequencing, and enhanced POC technologies with improved performance characteristics. Interestingly, the same is not necessarily true for nucleic acids (DNA, RNA), where saliva may provide a richer sample of target moiety than blood or plasma, depending upon the method of collection.

Saliva can also be influenced by confounds specific to the oral cavity. Recent eating can change flow rate and can also influence the rate or manner in which the salivary glands excrete fluid to initiate the digestion process. Excessive drinking can also influence some biomarkers, especially those which are actively transported rather than entering saliva via passive diffusion. Common confounds in saliva collection are food and drink particulates and residue, which are typically addressed in saliva collection protocols by requiring participants to rinse their mouths. Waiting approximately 5–10 min after taking a drink of water ensures that the sample has not been diluted by recent hydration. Given that some participants may find saliva collection difficult, prior research called for ingestion of acidic substances to enhance flow rates; unfortunately, this can potentially change the pH of the sample (44), influence the concentration of specific analytes in saliva, and render some biomarkers contaminated. Additionally, we have found that even the thought or smell of food can enhance salivary flow rates (45).

There is an obvious benefit in using saliva as a biofluid since it contains electrolytes, proteins, DNA, RNA, and lipids as well as an incredibly diverse variety of microbial species present in an individual’s saliva (30). Yet, the benefit of such a diverse mixture comes with drawbacks. The challenge is that, while not a class II biohazard, saliva is not a *sterile* biofluid: viral, bacterial, and food particulates may contaminate a saliva sample. There may be problems with contaminants in unique populations (e.g., milk within infants and neonates (46)) as well as distinct contaminants in other populations (e.g., alfalfa sprigs in horse saliva). Typically, the potential of particulates can make an additional filtration step or centrifugation process necessary, and meticulous pipetting techniques are required in standard enzyme immunoassay (EIA) protocols.

Saliva can also be influenced by characteristics of the individual that can impact individual salivary flow, such as exercise and stress, which slow salivary flow rate (47, 48). The autonomic nervous system, a main component of the stress response, innervates salivary glands in order to speed up saliva flow (and aid in digestion) during periods of rest through the parasympathetic nervous system (47). Conversely, the sympathetic nervous system also innervates salivary glands and slows saliva flow toward more crucial physiological functions during stress (49). This means that physiologically saliva flow slows and potentially changes its physiological makeup during periods of stress relative to “resting” saliva.

In addition to the effect the sympathetic and parasympathetic systems have on saliva secretion, there are two ways of collecting saliva specimens, based on salivary flow. *Unstimulated* saliva is typically collected *via* passive-drool, which involves pooling saliva in the mouth and collecting by expectorating directly or through a straw into a specimen tube. Conversely, saliva flow can be *stimulated* by chewing gum (50), swab-like collection devices, which may or may not contain citric acid (51), or sucking on a lozenge (45). The drawbacks of stimulating saliva flow include changes in pH (50), contamination of specimens, as well as potentially compromising biomarkers in the specimen (52). On the other hand, unstimulated saliva may also be contaminated with food particles, and may at times be too viscous, which presents additional difficulty during analysis.

Following on from this, the crucial confound is *viscosity*. Whereas other biofluids are more uniformly liquid, saliva can range significantly in terms of viscosity, the quantity of particulates, and the presence of bubbles (e.g., collecting specimens *via* passive drool method). Viscosity of saliva can vary from participant to participant and again the composition of saliva can also be influenced by exercise, stress, or hydration status. Our experience is that the collection time of the first morning saliva sample often takes longer and yields a more viscous sample than saliva collected later in the day. Viscosity concerns may also be specific to the individual. For example, patients with xerostomia (i.e., dry mouth) or patients prescribed with certain medications (e.g., antidepressants) can generate reduced salivary flow and increased viscosity. In instances where saliva may be too viscous, pad collection can help reduce the negative effects of viscosity by selectively binding mucins, which can interfere with downstream analysis. At a minimum, protocols should record the time of duration of saliva collection to potentially correct for saliva flow rates. Laboratory technicians should be properly trained to pipette diverse saliva specimens.

Last, depending on the biomarker of interest, the choice of collection device must be carefully considered, as the materials used in some collection devices can interfere with saliva for some biomarkers, but not others (52). To illustrate the importance of careful consideration of the collection device, consider the breadth of attention paid to the design of saliva collection devices. The first saliva collection devices were approved by the FDA in the 1990s when OraSure Technologies gained FDA 510(k) approval for the OraSure Saliva Collection Device (OraSure Technologies, Bethlehem, PA, USA). Around the same time, the Saliva Sampler Collection Device, developed by Saliva Diagnostic Systems (Saliva Diagnostic Systems, Framingham, MA, USA) also gained FDA clearance. Another early saliva collection device on the market was the Sarstedt Salivette (Sarstedt, Numbrecht, Germany), which is commonly used in research studies, but has not been FDA approved. Many other devices have been developed to fit the growing interest and need for proper saliva sample collection [for a detailed overview of saliva collection devices, see Ref. (53, 54)]. Across collection devices and between stimulated and unstimulated saliva, there can be significant differences in the concentrations of analytes quantified, so it is important to determine both the most viable collection methodology and technology required, depending upon the biomarker of interest and the specific population under evaluation.

Another factor to consider is the use of preservatives such as sodium azide, which are essential for the stabilization of certain biomarkers (e.g., proteins). However, it is important to be aware that the inclusion of such reagents can invalidate certain methods (e.g., EIA protocols for salivary cortisol, Salimetrics, PA, USA) that rely on precise biomarker concentrations to quantitate sample values. This potential drawback may not be realized until after sample collection if, for example, additional biomarker assessments are desired following study completion. Failure to properly consider the unique challenges of saliva have adversely impacted its viability in the past, yet, careful consideration of the potential challenges highlighted here should allow this biofluid to continue to be valid and reliable. All bodily fluids have inherent challenges that should be considered thoughtfully throughout the design and implementation of collection and analysis protocols.

## Salivary Cortisol

Given the wide range of biomarkers that are detectable across biofluids, it is beyond the scope of this paper to exhaustively review all of the possible applications, so, for this reason, this section focuses specifically on salivary cortisol for both practical and historical reasons [for additional information on saliva as a diagnostic biofluid, see Ref. (55)]. A practical justification is that salivary cortisol provides a precedent and strong scientific reasoning for why detection of the biologically active fraction of a hormone in oral specimens is preferable to analysis using other bodily fluids. Combined with the advantages of non-invasive stress-free collection, cost effectiveness, and high patient compliance and preference, salivary cortisol demonstrates unique advantages over blood. These and other features have resulted in a burgeoning interest in salivary cortisol research and clinical applications for the hormone. Historically, salivary cortisol was one of the first salivary biomarkers to achieve widespread acceptance within the research field providing the basis for evaluation of both strengths and weaknesses of a specific salivary biomarker. Nonetheless, cortisol researchers were also the first to recognize major concerns with collection methods that altered the pH of a sample and threatened to invalidate years of published research (44).

### Cortisol

Commonly known as the “stress” hormone, *cortisol* is a product of the hypothalamic–pituitary–adrenal (HPA) axis. In response to changes in the environment (i.e., “stressors”), the brain (largely limbic and other emotion-related neural structures) initiates the HPA axis. This hormone cascade begins within seconds in the hypothalamus, stimulating it to release corticotropic-releasing hormone (CRH), which acts as a stress neurotransmitter within the brain, also acting as a hormone that travels through a small limited blood supply to the anterior pituitary. CRH signals the pituitary gland to release adrenocorticotropic-releasing hormone (ACTH), which travels throughout the circulation to bind to receptors within the cortex of the adrenal glands. ACTH triggers the release of several stress hormones into the blood, but the most abundant in humans is cortisol. This hormone is released from the adrenal glands within minutes of exposure to a stressor and peaks in concentration within about 15 min of a stressor onset.

The time course of cortisol is important to note as its release is initiated within seconds, reactivity occurs across minutes, and yet the physiological impact of cortisol can last hours to days. The release of cortisol is regulated within the body through, for example, the circadian rhythm or the cortisol awakening response, which helps the individual prepare for the day ahead (56, 57). Cortisol release can also be changed in response to a stressor, such as when unpredictable social contexts or social evaluative threat stimulate cortisol reactivity (58, 59).

The social context for cortisol collection is also highly important to consider. Cortisol reactivity can be stimulated within the laboratory, clinical, or research setting where it is also feasible to collect blood. However, venipuncture itself triggers a cortisol response (60). To minimize the effect of the induced stressor, samples must be collected very rapidly prior to initiation of a stress response, through use of an intravenous catheter, or with an ample acclimation period so that the reactivity induced by venipuncture can return to baseline (61). Cortisol reactivity is also observed in response to naturalistic stressors (62), sometimes at much higher levels than would be ethical within a laboratory, and where venipuncture is impractical. For example, skydiving triggers greater cortisol reactivity than even the most highly validated laboratory stressors (63).

The physical properties of cortisol have also been a contributory factor in the exponential growth of salivary cortisol research. Cortisol is a hydrophobic steroid hormone, which is largely bound in the blood to bulky carrier proteins (i.e., cortisol binding globulins) that permits it to flow throughout the circulatory system. The highest concentrations of cortisol are found in blood (61), but a small portion is unbound and lipid-soluble and thus capable of passing through the double-lipid cell membranes around the body (64). This unbound cortisol is the biologically-active fraction due to its ability to pass through the blood-brain barrier to directly influence neural functioning, or through cell and nucleic membranes to directly change gene expression (65). Importantly for saliva, it is this unbound or “free” fraction which also passes through the acinar cells to enter saliva via passive diffusion.

The general role of cortisol is to facilitate fight-or-flight response, in particular the longer duration of the stress response in which the body must sustain activity through enhanced glucose metabolism. Cortisol also functions through sustained counter-regulatory mechanisms to terminate a wide range of initial components of the stress response. Cortisol terminates the stress response through negative feedback, by inhibiting further CRH release. Cortisol also reduces the fight-or-flight physiological response to acute stress, and exerts anti-inflammatory effects on immune functioning. Physiological effects of cortisol can last hours or days, even long after cortisol concentrations return to baseline via the functional role of cortisol in epigenetic and genetic regulation.

In this section, we use the stress hormone cortisol as an example biomarker since cortisol nicely illustrates the strengths and weaknesses of saliva as a biofluid. The non-invasive nature of saliva, the advantages of this medium for POC testing, and a movement to collect specimens remotely and transport samples to a centralized location for laboratory testing, have allowed saliva to become the “gold standard” for cortisol quantification in the research environment. The time course of a stress response has thus far been considered largely from the vantage point that protocols call for precise timing of saliva collection and the field has a very well-characterized understanding of the time-dependent fluctuations of cortisol. What has not been addressed to this point is that in current practice, saliva samples are typically frozen and cortisol assayed days to weeks after collection. To take full advantage of the benefits that saliva provides, rapid, sensitive, specific, accurate, and cost-effective analytical methods are needed. Below we present a newly developed alternative assay method for the efficient collection of saliva and subsequent POC analysis for cortisol, and potentially other biomarkers. We review briefly how near real-time assays operate, with focus on the potential of LFIA application to be adapted to a saliva specimen. We return to cortisol as an example biomarker given that it illustrates the potential for real-time biomarker measurement to impact the field.

## Lateral Flow Immunoassays

There are many test formats used for rapid (i.e., within minutes) testing, but in our synopsis below, we confine our discussion to LFIAs, which are specifically designed for use in POC settings. LFIAs have been around for a long time and many examples of LFIAs exist. There are only a handful that use saliva specimens, particularly within the research market. Therefore, it is difficult to carry out a direct and thorough strengths and weaknesses analysis. Instead, we illustrate the strengths and weaknesses by comparing LFIA to more commonly used research assays—(EIA)—so that the reader can evaluate the new LFIA technology in an informed manner.

### Overview of LFIAs

**Figure 1:**
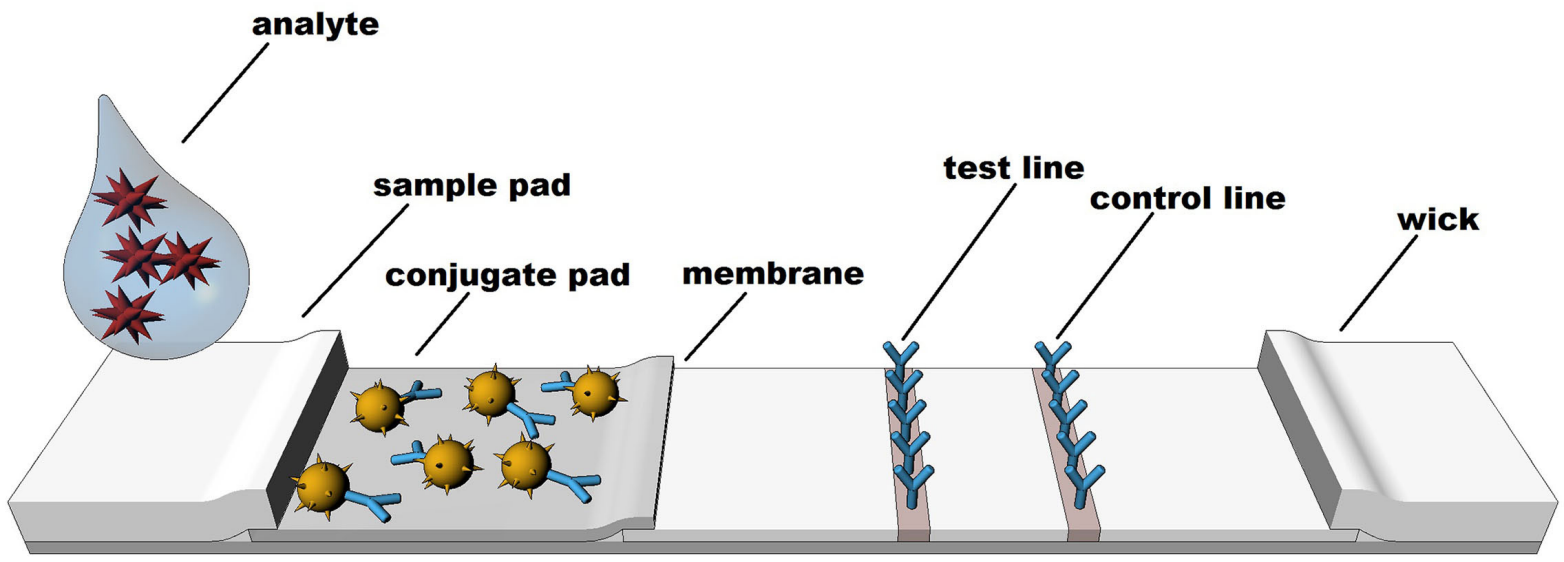
Structure and sequence of a typical lateral flow test strip.

Rapid diagnostic tests are based on technologies which rely on a visible color or instrument detected change to identify the presence or absence of specific molecules. Although other formats exist, LFIA techniques such as colloidal gold, latex, or various other particle-based systems are the most common formats (66). LFIA rapid diagnostic testing works on the basis of liquid movement across a strip of polymeric material containing dry reagents that are activated by the lateral movement of a liquid sample up the strip membrane. The LFIA strips contain specific sections with antibodies to which the analyte will or will not bind, depending upon the format and biomarker of interest (see Figure 1). Binding of the analyte to the antibody strips results in a detectable change (e.g., color or fluorescence) that is directly related to the amount of analyte bound on the strip. A control line on the strip indicates proper function of the test. In similar fashion to traditional EIA technology, the two most common formats for LFIAs are sandwich (large molecule detection) or competitive assays (small molecule detection), which functionally rely on color change to detect an analyte.

Perhaps the most well-known examples of LFIAs include over-the-counter lateral flow pregnancy or fertility tests and HIV tests, which are available for testing in the privacy of one’s home. Pregnancy and fertility are assessed by detection of the hormones human chorionic gonadotropin, luteinizing hormone, or follicle-stimulating hormone in urine to indicate a physiological state. Pregnancy and fertility tests illustrate the long history of hormonal measurement with LFIAs. HIV tests on the other hand detect antibodies to the HIV virus, which are detectable at sufficient levels in biological fluids, including saliva. LFIA technologies have been largely qualitative in nature, yet quantitative LFIAs represent a highly interesting potential for screening and monitoring applications across a wide range of diagnostic areas. The present paper explores the potential for salivary LFIA to experience continued growth in both the number and variety of applications, including development and adaptation of quantitative assessment and new biomarkers (e.g., cortisol).

Common LFIA systems on the market readily illustrate the advantages of the technology in non-traditional environments outside the laboratory. These advantages of rapid diagnostics include (a) Reduced turnaround time to receive results; (b) Collection and delivery of results within POC settings; (c) Potential reduction in the cost of specimen collection, storage, transportation and processing; (d) Elimination of the need for the patient to return for results and (e) Increased communication between the researcher or clinician and the participant, as results can be discussed at the time of testing. LFIA exhibits these and other advantages, which allow for testing and returning results largely by completing much of the assay process before the end-user confronts a lateral flow test strip.

### The Underlying Technology behind LFIAs

Perhaps the most advantageous characteristic of LFIA is its simplicity, from the user’s perspective. The apparent simplicity, however, masks an extensive development and manufacturing process, which involves highly advanced techniques that occur “*behind the scenes*.” This “internal development” process by manufacturers to produce LFIA technology (67) renders the technology easy for the user to carry out and interpret (68). New developments in manufacturing technology and process control over the last 10 years have allowed LFIA technologies to flourish. Prior to that, LFIA technologies were difficult to reproduce on a consistent lot-to-lot basis. Development of LFIA test strips requires careful choice of the appropriate antibodies for pairing, which may vary depending on the specimen matrix of interest (i.e., saliva, urine, or blood) and typically involves extensive trial and error to find the best affinity for the specified test. LFIA scientists must define the best signal to use to produce well-defined results (68), with common options including colloidal gold or carbon, fluorescent or luminescent materials, or colored latex beads. As an example, color from colloidal gold nanoparticles generates a direct signal, whereas use of other materials may require additional steps in order to derive analytical results. Selection of the best detection technology for LFIA must consider whether LFIA materials provide optimal performance for the specific analytes under investigation, and must balance this performance expectation with the user’s preference for required turnaround time. The development of lateral flow test strips also requires that LFIA experts consider the composition of the fluid sample that will flow up the strip, whilst also accounting for potential variation in the characteristics of the fluid sample (particularly viscosity). LFIA designers must also anticipate the potential confound of the user themselves, often taking strides to ensure that the end-user cannot interface directly with the test strips or alter the flow of fluid along the membrane. To protect against this possibility, most LFIA cartridges are robust, plastic, single-use designs without ready access to LFIA strips. The LFIA expert must configure the optimal design of a housing cartridge for the test strips to meet the anticipated needs of the market where the product/technology will be sold (e.g. over-the counter, versus testing in low resource settings, in the field and other POC locations).

Once the LFIA is developed, the process continues into the manufacturing phase, performed in controlled conditions, particularly temperature and moisture. Test strips are sprayed, conjugate pad is applied, sample and absorbent pads are added, and then strips are cut to the desired width and length. Test strips are assembled in a sandwich format (see (68)) and then sealed into well-designed cartridges that protect the test strips from interference or positional variation. Manufacturers must ensure that *only* the molecules of interest bind to the antigens or antibodies coated onto the tests strips, whilst also ensuring the sample matrix is sufficiently purified for direct application to the test strips without interference from aggregation factors and other interfering molecules found in bodily fluids. The cartridge that contains the test strips is often a highly engineered piece of plastic that ensures accurate delivery of specimens and smooth test functioning. Precise manufacturing of the housing cartridge must be carried out consistently and on a lot-to-lot basis. Beyond initial 3D printed prototypes, the LFIA expert must have access to expensive equipment for precise and reproducible large scale-production of the LFIA components (see (67)). This includes equipment such as plastic injection molds, sonic welding equipment, high throughput assembly and packaging equipment, as well as others to ensure a high quality product that meets the requirements for robust testing by multiple populations.

### How Does LFIA Differ from EIA?

In comparison to the more established EIA technology, LFIA has relatively few players. Early lateral flow tests started to appear in the late 1980s and early 1990s, yet LFIA technologies remain quite novel. Multiple platforms exist for LFIA using different molecular targets found in multiple fluids (serum, whole blood or urine), however, relatively few use saliva and even fewer can boast high sensitivity or full biomarker quantification. The ability to perform fully quantitative analyte assessment is restricted to a handful of companies with specific expertise in the area. The current landscape is dominated by LFIA technologies that are controlled by commercial companies or academic institutions with strong intellectual property portfolios. In many cases, these technologies may be public domain or openly licensed for new diagnostic tests, leading to an increase in available LFIA-based assays. LFIA’s comparatively short track record has also allowed there to be significant variability in the cost of LFIA devices, cartridges, and (if quantitative) LFIA readers. Whereas EIAs can be created through “in-house” technology, many of the innovations within the LFIA field are more commonly developed by commercial entities. Devices and the technologies upon which they are developed may be patentable, leading to licensing opportunities for third parties. While commercial interest can lead to faster technology development, under certain circumstances, information may be held as “trade secret,” which benefits the technology developer, but can stifle widespread application. Given specialized equipment and knowledge, ideas developed within the academic community must typically interface with industry experts to ensure adequate quality control (QC) requirements are met. Moreover, empirical studies on current quantitative lateral flow tests are scarce in order to protect intellectual property, which results in skepticism from the academic and scientific community until tests have been fully validated through reproducible research.

The “secretive” nature of LFIA makes it difficult for the curious end-user or the diligent researcher to understand and evaluate the technical capabilities of LFIA. To address this, the following section provides a comparison of LFIA with EIA so that the knowledgeable reader can apply their base knowledge about immunoassays to better understand the capabilities of LFIA. In comparing the two techniques, the goal is not to suggest that LFIA will replace EIA, but rather act as an “adjunct” to laboratory testing using EIA technology. It is relevant to explore the potential environments where LFIA can provide additional information to aid the clinician, researcher, healthcare worker, or patient by providing rapid turnaround of results.

Enzyme immunoassay and LFIA differ in terms of *turnaround time*. This divergence begins with sample preparation. EIAs commonly require freezing, thawing, and further preparing samples (i.e., vortexing and centrifuging), and then precisely pipetting specimens and chemical reagents into each well of a 96-well microplate. Most EIAs minimally require several hours to perform under optimal conditions and are performed in batch mode in order to complete a full 96-well plate at a single time. Batching to obtain sufficient samples to make the analysis cost effective adds significant turnaround time for results, and hours to days to weeks can typically pass between receipt of a sample and returning an EIA result. The turnaround time is much faster with LFIA, which can be done individually (often while the subject or patient is still present) in STAT mode with turnaround of results within minutes based on an individual sample rather than a full or partial EIA plate.

There are significant *procedural differences* between EIAs and LFIAs. Each 96-well EIA plate involves many preparatory procedural steps which involve specialized laboratory training and equipment in order to reduce user-related sources of error. In addition to following a strict protocol, EIAs are time-sensitive, with highly specific incubation times in between addition of each reagent. EIAs are consequently classified as high complexity, according to CLIA (69). End-user procedural errors are less likely with LFIA because, as described above, much of the test process is performed “behind the scenes” and masked from the end-user. LFIAs require fewer steps and significantly less user training. Consequently, many LFIAs are considered low complexity. Indeed, a number of them (including the OraQuick HIV 1/2 test for oral fluid testing for the HIV virus) have been given a CLIA-waived status which allows them to be performed by “untrained” users.

Enzyme immunoassay and LFIA differ in terms of *workflow*. EIA protocols often include multiple wash cycles of the 96-well microplates to remove all unbound residue from the biofluid except for the bound analyte. Wash protocols are in addition to sample preparation steps and must be precisely completed to ensure that final EIA test results are not negatively impacted by sources of error introduced during the wash cycles. In contrast, LFIAs do not have wash steps and must be designed to accommodate “crude” biofluids, which are not highly purified prior to analysis, or LFIAs may need to include a simple, fast, and short purification step to ensure a clean specimen reaches the LFIA test strips. For LFIA tests to perform satisfactorily, crude or cleaned up biofluids must produce discreet bands on the lateral flow test strips at well-defined *Test* and *Control* regions. In order to accomplish this, LFIA manufacturers must compensate for the unbound residue remaining on the strips, without affecting test results.

End-users of EIAs require large and specialized equipment to obtain results. The laboratory must be equipped with instrumentation for storage and processing of samples (e.g., centrifuge, vortex, ultra-freezer, and standard refrigerator), and assay-specific equipment for 96-well plates (e.g., plate washer, plate reader, biosafety cabinet, precision pipettes, vortex, plate shaker, etc.). In some cases, additional lab equipment may be required (e.g., autoclave, pH meter), which are expensive but generally available in a biosafety level II laboratory. Alternatively, samples can be shipped to a biosafety level II laboratory, but this does not circumvent the need for storage facilities (e.g., freezers) and shipping supplies (e.g., cool packs or dry ice), which add to the cost. This contrasts with minimal LFIA end-user equipment requirements. LFIAs typically do not require equipment such as centrifuges for sample processing or ultra-freezers for sample storage. Most LFIAs can be stored at room temperature for long periods of time prior to sample processing, provided that devices are retained in their original packaging to protect against any moisture intake. Qualitative or semi-quantitative LFIAs (e.g., pregnancy tests, rapid HIV tests, fertility tests) do not require any reading equipment to interpret test results. These tests are purely visual and read easily by untrained users.

The newest generation of LFIAs have been improved to the point where full quantification is a reality, although quantitative LFIAs require “readers” that contain image analysis computer software and specialized instrumentation that detects particles on the test strips (fluorescence, chemiluminescence, etc.). Readers for quantifying LFIA strips are not generic like typical EIA microplate readers that measure optical density in order to determine biomarker concentrations. Instead, quantitative LFIA readers differ in the type of signal they detect (i.e., fluorescence or color), the particular environment the test strip is in (e.g., cartridge format), number of test strips read, and programming within the reader specific to the test format. Manufacturing an LFIA reader requires inputs for design of both the reader and the analysis software. Many manufacturers of quantitative lateral flow tests design and manufacture test or technology specific readers that are highly specific to their tests.

Although both EIA and LFIA putatively rely on “color change” to detect analytes, they differ in how they *convert results into analyte concentrations*. In order to establish analyte concentrations, EIAs require a standard curve to be constructed using the optical density values of calibrated materials and then the optical density values of samples are interpolated relative to the standard curve concentrations. For each 96-well plate, this standard curve is recapitulated and a certain number of control wells are dedicated to validating each independent sample run. Samples are typically assayed in duplicate or triplicate to improve precision and identify erroneous samples that must be rerun at a later date. These duplicate wells also permit calculation of the coefficient of variation (CV) for the assay. LFIAs, in contrast, calculate conversion to analyte concentrations based upon quality control (QC) protocols during the manufacturing process so such validation steps are not performed by the end-user. Replication of an erroneous LFIA, if desired, is typically accomplished by having the end-user repeat sample collection on a fresh device. Recent advances in LFIA improve quantitative LFIA results by testing side-by-side duplicate test strips in a proprietary housing, much like an EIA protocol. The average of the duplicate LFIA results is reported by the reader in order to increase precision over single strip systems.

## Recent Advances in LFIA Technology

Lateral flow immunoassays are relatively new considering the extensive history of the *in vitro diagnostics* business, but the field nonetheless continues to innovate and several advances have emerged within this novel field in recent years. There have been exciting technological advancements and improvements in the manufacturing of rapid, small-scale tests. For example, lab-on-a-chip (LOC) systems have a number of positive attributes including convenience, compact format and large scale manufacturability, in addition to providing a practical solution for laboratory operations to be done on a smaller scale and in close proximity to the patient (70, 71). LOC systems are integrated microfluidic devices, which look like a computer chip with microchannels. These microchips are not considered lateral flow devices (LFDs), rather they work on the basis of microfluidics with a sensor for detecting the analyte. LOC systems require a reader that interprets the signal from the chip (70), and are used for detection of pathogens such as a variety of E. coli, H1N1, Noroviruses (71), as well as DNA analysis (72). Another example is paper-based tests, which are designed to process very small volumes (i.e., nanoliters, and picoliters) of liquid samples (73, 74). Paper-based microfluidic tests began in the form of traditional dipstick tests, designed to quantify glucose in the urine of diabetes patients, returning a form of semi-quantitative result interpretation (53).

A second recent advance in rapid testing technology is illustrated by the innovation in salivary LFIA applications. Episcreen became part of the first clinically adopted saliva test for HIV following FDA clearance in 1997. The Episcreen device, now branded under the “OraSure” name, is a standardized device for saliva collection used in conjunction with an EIA kit from Organon Teknika (Boxtel, Netherlands) as part of the first laboratory-based oral HIV test. Several years later, the manufacturer of the OraSure device (OraSure Technologies) developed the world’s first oral based *rapid* LFIA HIV test. This test, known as the OraQuick HIV 1/2 rapid antibody test, was approved by the FDA in 2005, and later for over-the-counter applications in 2012. Salivary LFIAs have also penetrated the market in the area of drugs of abuse testing around the world and several systems have been FDA cleared for marketing (75), although testing remains largely qualitative in nature. Salivary rapid tests have also been developed for infectious diseases (see (76)) and the company SOMA Bioscience (Wallingford, UK; previously iPRO Interactive) has developed quantitative saliva LFIAs for IgG, IgA, α-amylase, and cortisol. Their salivary cortisol LFDs have been used in sports research (34, 77, 78). Below we focus on real-time cortisol as this analyte has the most published scientific literature to date as a quantitative LFIA.

### Rapid Assessment of Salivary Cortisol

There is substantial potential for growth in the industry using saliva for LFIA, but it is difficult to determine whether increasing interest and availability of gadgets for POC diagnostics is warranted. In view of this statement, a brief review of the field for salivary cortisol LFIA is presented. Rapid tests can assess salivary cortisol within minutes instead of hours and we note that there are such tests in one form or another that have been available to the scientific community for some time.

Over a decade ago, a dipstick for quasi-quantitative assessment of cortisol concentrations in plasma was developed by Leung and colleagues (79). The dipstick test is designed similarly to other LFIAs, which rely on a liquid sample flowing across a test strip containing bound antibodies. Time-to-result is less than 5 min, and the intensity of color development on the test strip is directly proportional to the cortisol concentration. The results are estimated visually or can be analyzed quantitatively with a personal analyzer for rapid tests (PART). This technology had limited application and was not widely adopted because it required plasma specimens, as well as potentially because the quantitative determination was questionable.

A decade later, other LFIA cortisol devices (see Table 1) began to appear although it is difficult to determine whether these devices reached commercialization or are still at the conceptual stages. Yamaguchi and colleagues (80) developed a cortisol immunosensor for quantitative assessment of salivary cortisol. The novel design of this immunosensor incorporated a mechanism that controlled both vertical and lateral flow. Shirtcliff and colleagues (81) developed a salivary cortisol assessment system (VerOFy^®^), which includes a saliva collection device and filter system, duplicate lateral flow strips for increased accuracy, and a reading unit- the Litebox Image Analysis Module (LIAM™) with integrated analysis software to quantitate cortisol levels. The salivary cortisol lateral flow test developed by Dunbar et al. (82) includes an IPRO oral fluid collector (OFC), a LFD and an IPRO LFD reader.

**Table 1:** Salivary cortisol lateral flow immunoassays (LFIAs).

Salivary LFIA technology	Components of system	Sample collection method and preparation	Time	Range of cortisol values (ng/mL)	PROS	CONS
Salivary cortisol immunosensor Yamaguchi et al. (80)	Immunosensor	Sample is mixed with conjugate, phosphate-buffered saline and glucose solution	35 min	0.1–10 ng/ml	– Innovative design and salivary cortisol measurement system – Uses detection of current to determine cortisol level	– Extensive sample preparation and analysis process, no guidelines
VerOFy^®^ Salivary Cortisol Assessment System With LIAM™ Shirtcliff et al. (81, 89)	– VerOFy^®^ saliva collection device and cartridge with two LFT strips. – LIAM™ reader with Bluetooth connectivity	Sample is collected *via* VerOFy^®^ collection device. Sample is filtered then saliva is dropped into duplicate wells of LFT cartridge	20 min	0.5–25 ng/ml	– No sample preparation (i.e., buffer or conjugate) – LIAM™ reader with Bluetooth compatibility – Duplicate testing for increased precision	– Requires a system-specific reader
Stress measurement smartphone system Choi et al. (83)	– Smartphone holder – Lateral flow test strip – Buffer	Sample is collected *via* swab, mixed with buffer, then dropped onto lateral flow test strips	10 min	1–100 ng/ml	– Smartphone acts as reader of LFT strip – Large range of detectable values	– Requires smartphone camera calibration. Likely to require frequent updates as phone software changes
Smartphone chemiluminescence-based salivary cortisol LFIA Zangheri et al. (84)	– Smartphone with adaptor – Additional lens – LFT cartridge – Conjugate and substrate	Sample is collected *via* swab, expressed and added by syringe to cartridge containing prefilled solution of conjugate and buffer	25 min	0.3–60 ng/ml	– Smartphone acts as reader of LFT strip – Large range of detectable values	– Extensive sample preparation and analysis process – Smartphone cameras, software may vary (as above)
Cortisol lateral flow device (LFD) (previously iPRO sCORT POC LFD) Dunbar et al. (82)	– Oral fluid collector (OFC) with buffer – LFD with reader	Sample is collected via OFC, mixed with buffer, then dropped onto LFT strip	12 min	0.75–15 ng/mL	– Easy and rapid test – Commercially available	– Requires system-specific reader

Others also took advantage of the POC potential for salivary LFIA by augmenting LFIA with smartphone connectivity. Choi et al. (83) developed a “stress measurement” (i.e., cortisol) smartphone system comprising of a smartphone, holder, and a lateral flow test strip and Zangheri and colleagues (84) also developed a smartphone-based chemiluminescence LFIA. This latter system consists of the smartphone, which serves as a light detector, a 3D-printed cartridge housing the lateral flow strip, as well as a smartphone adaptor with an additional lens, also designed to house the LFIA cartridge.

These devices differ in terms of technique for sample preparation. Shirtcliff and colleagues (81) clean the sample through the use of a patented collection device as well as a unique proprietary filter designed to capture food particles, normalize saliva viscosity, reduce bubbles, and break down large protein molecules prior to LFIA. This filtration system eliminates the need for dilution, freeze/thaw, or centrifugation while still returning a uniform saliva sample, largely free from interfering mucinous materials. Choi and colleagues (83) and Dunbar and colleagues (82) purify the sample by requiring the user to collect saliva with a swab, which is then mixed with buffer and dropped onto the test strip. Zangheri and colleagues (84) system consists of a chemiluminescent biosensor, which requires a washing step using phosphate-buffered saline (PBS), followed by addition of a chemiluminescent substrate for preparation and development of the sample. As noted above, saliva is a challenging biofluid and users may run into difficulty with the presence of bubbles or the viscosity of different saliva samples. In addition, cortisol concentrations are low in saliva and dilution may be problematic to keep samples within the detectable range. Some of the above systems do require specimen dilution (84) or provide a buffer for dilution of the sample (82, 83).

Potential users should also consider the range of detected cortisol values. For instance, the smartphone system described by Choi et al. (83) captures salivary cortisol values between 1 and 100 ng/mL and Yamaguchi et al. (80) captures values in the range of 0.1–10 ng/mL. The LFIA system for Zangheri et al. (84) captures cortisol values in the range of 0.3–60 ng/mL, whereas Shirtcliff et al. (81) detects salivary cortisol values between <0.5 and 25 ng/mL. Dunbar and colleagues’ (82) LFD reader detects values between 0.5 and 15 ng/mL. A low detection limit and a wide range of detectable concentrations is important for cortisol due to the ability of cortisol to change within minutes as well as the well-reported diurnal fluctuation throughout the day [i.e., commonly high in AM and low in PM; see Ref. (85)].

Another consideration is the read time for the assay. Turnaround times for result reporting range from the technology returning values within 10 min (83), or 12 min from sample collection (82) to technology returning values within 20 min (81), 25 min [including saliva flow and washing step (84)], or 35 min (80). It takes 15–20 min for cortisol to peak after a stressor (86); therefore, the average time to delay of results across technology suits the time course of cortisol release and delays of minutes can be considered “real-time” particularly when contrasted with EIA delays spanning hours to months. Nonetheless, other stress biomarkers with faster reactivity profiles (e.g., alpha-amylase) may need to quantitate results much faster to be considered “real-time.”

A more nuanced consideration is the reliability of the method of developing the salivary cortisol assay. Choi and colleagues (83) smartphone system requires the user to insert a smartphone into the provided holder. The smartphone uses internal imaging capacities to take a photo of the LFIA test strip. The smartphone application is equipped with an algorithm, which detects the lateral flow strip, and through changes in hue and brightness calculates a cortisol value. Zangheri and colleagues (84) also use a smartphone as a light detector as well as a smartphone adaptor with an additional lens in their chemiluminescent biosensor. Following a PBS washing step and addition of a chemiluminescent substrate, the cartridge is inserted into the smartphone adaptor, and chemiluminescent signals are acquired through the smartphone camera. Concerned with the variability in camera specifications across smartphone manufacturers and operating systems, Shirtcliff and colleagues (81) produced a robust and reproducible imaging method using a separate (LIAM™) reader, which was specifically designed and upgraded in development to improve capturing and analysis of cortisol binding on lateral flow strips. Dunbar and colleagues’ (82) system has a fluorescent (LFD) reader.

Lastly, each technology must be evaluated according to the particular needs of the end user. Smartphone compatibility emphasizes the tech-savvy end-user (83, 84), however, these may be more difficult to assess for reliability and reproducibility of quantitated cortisol. Technology described by Yamaguchi and colleagues (80) may be difficult to implement (given sample processing requirements) for a novice end user. Shirtcliff and colleagues (81) specifically designed technology for research-oriented applications by maximizing compatibility with EIA technology through three ways. The filtered sample remaining following analysis can be used for confirmation *via* EIA. The cartridge contains two lateral flow test strips, similar to EIA duplicate testing. Finally, the LIAM™ reader is tightly calibrated through internal imaging capacity and software algorithms for each lot. The smart-phone interface is accomplished after reliable results are read. Dunbar and colleagues (82) offer a salivary cortisol assessment system that is easy to use with rapid turnaround of results, reported to an “off-the-shelf” reading device.

Across these cortisol assessment systems, there are differences in design, preparation of samples (e.g., use of buffer or conjugate), and lateral flow strip manufacturing. The point is not to advocate for a specific technology, but rather to illustrate that the field is ready to consider novel LFIA technology such as salivary cortisol LFIA. This enthusiasm must be reinforced through careful consideration of each technology’s strengths and weaknesses.

Although we focus our review on salivary cortisol, it is notable that the apparent advantages of LFIAs has also resulted in an expansion of the range of LFIAs now commercially available involving different fluids. For example, test development has been applied to a wide variety of analytes and diseases including HIV, rubella, syphilis, hepatitis, drugs of abuse, and the presence or absence of toxic and infectious compounds in food or liquids, among others. These advantages are expected to be captured by multiple commercial concerns, which in turn will result in a significant growth in the field.

Rapid salivary LFIAs also have the potential application in the future to many other salivary biomarkers and combinations of biomarkers. For example, testosterone and dehydroepiandrosterone (DHEA) are responsive to stress, vary in concentration across short periods of time, and are, therefore, time sensitive. Testosterone is crucial in research and clinical settings, in the area of competitive sports and physical activity, as well as in aggression. DHEA is a useful biomarker associated with aging (87) and treatment of depression (88). Future expansion of the LFIA market is likely to include other small molecule analytes such as testosterone and DHEA, which in turn will be important for the advancement of stress and developmental research and will potentially have application in a wide variety of fields of use, including sports medicine, aging, reproduction, etc.

Beyond the development of LFIAs for these specific hormones, salivary LFIA technology in general offers great potential for the future development of tests assessing multiple biomarkers in simultaneous fashion, within minutes of sample collection, and at no risk to the donor. Biomarker panels are made more feasible through the integrated reader strategy to obtain results through software algorithms developed within reading devices that facilitate interpretation of results from biomarker panels. A rapid multiple-biomarker panel can be versatile enough to fit specific needs of patients across clinical, research, and industrial contexts. In addition to providing the convenience and simplicity of a rapid salivary LFIA to the user, this technology can allow the users’ individualized insight into their own health and body function. Such biomarker panels will likely rely on a larger existing saliva-based body of literature to guide algorithms and advance interpretation of how multiple analytes work together (89).

## Conclusion

This paper provides an overview of the current state of salivary biomarker detection using lateral flow technology and highlights the advantages and disadvantages of manufacturing and using LFIAs for the purpose of salivary biomarker detection and analysis. Although quantitative salivary LFIAs are still in their infancy, the growing interest in rapid salivary biomarker assessment described above illustrates the enormous potential this technology has to improve the fields of diagnostics and research. The success of salivary cortisol for research and diagnostic purposes further illustrates the size of the field that can feasibly take advantage of the strengths of saliva, such as its non-invasive nature, applicability to remote collection and POC settings, and simplicity of collection. The additional benefit that LFIAs offer by returning near real-time results can be a critical additive factor in ensuring that the range of applications continues to grow exponentially. At this time, LFIAs using saliva have not been standardized or properly presented and evaluated by the academic audience. In order for the field of rapid testing and diagnostics to flourish, we encourage the diagnostics industry to establish collaborative partnerships with the academic community, and for lateral flow tests developed to be tested and evaluated with academic rigor.

## Author Contributions

OM is responsible for writing, editing, and literature review. CRC, MJL, RLB, PDS, and EAS contributed to writing, editing, and literature review.

## Conflict of Interest Statement

Two of the contributing authors, ES and PS, are research topic editors. Co-authors PS, RB, CC, and ML are affiliated with Oasis Diagnostics Corporation whose cortisol assessment device is described in the manuscript among other cortisol assessment systems. Furthermore, ES and Iowa State University have a subcontract with Oasis Diagnostics Corporation as a beta-testing site for the salivary cortisol assessment system. OM declares no conflict of interest.
